# Remarks upon Amputation at Hip Joint, with Case

**Published:** 1871-02

**Authors:** T. Mack

**Affiliations:** St. Catherines


ART. IV.—*Remarks upon Amputation at Hip Joint, with Case.* By Professor T. MACK, of St. Catherines.

The danger of death from shock being removed by anæsthesia, and the control of hemorrhage facilitated by compression of the abdominal aorta, the delay in disarticulation alone remains as a slight difficulty in the fifteen different methods proposed for this formidable piece of surgery. I am not anxious to add a sixteenth, but if the report of the following case shall be found to add to the fertility of resource, which is so valuable a qualification for the surgeon, I shall feel indemnified for the trouble of communicating it for publication.

John Connor, aged 7 years, of an eminently scrofulous appearance, was admitted into the General and Marine Hospital here on the 27th December, 1858, for morbus coxarius in the suppurative stage; several sinuses already existed, and, in a few days after his admission, an incision permitted the discharge of a large collection of unhealthy curdy pus. Under cod-liver oil, and appropriate constitutional treatment, he improved so much that it was decided to give him a chance for life by excision of the upper end of the femur.

On 7th July, 1869, having submitted him to the influence of chloroform, and assisted by my brother, Drs. F. Mack, Goodman, Sullivan and Comfort, I proceeded to the resection by making a semi-lunar incision, convexity downward, and extending farther down than directed by the books. The flaps being dissected and reflected upward, the joint was quickly reached and the capsular ligament cut through, when, at the moment the head of the bone started out by being gently everted as the shaft was adducted, the bone was fractured obliquely through the lower third. Inferring abnormal fragility from softening of osseous tissue, I immediately proceeded to amputation. An assistant having efficiently controlled the artery, I entered my knife upon the inner side of the disarticulated joint and cut out a sufficient internal flap, the femoral, ischiatic and obturator arteries were ligated, other spouting vessels being secured by torson. The boy was now allowed to awaken from anæsthesia; scarcely any blood was lost; the surfaces were exposed to the air until well glazed; all clots were carefully removed; stimulants were administered. He was again anæsthetized, the edges of the wound were united by silver sutures, weak carbolic dressings were applied; and, under the unremitting care of Drs. Goodman and Mack, he made an excellent recovery, so that, in three months, he was able, with a crutch, to go to school and to walk nearly a mile, only a slight discharge continuing, which was completely stopped about six or seven months after the operation. About a year had elapsed when an abscess again pointed; and, although I am informed that he made a fair recovery, still I have little doubt that the scrofulous disease in the pelvic bone will eventually claim its victim.

Before closing the wound an examination of the cotyloid cavity showed the bony structure in great part destroyed; the carious bone was carefully removed. The head of the femur was ulcerated away to fully one-half of its extent—the shaft of the bone a mere brittle shell—the medullary cavity filled with a puruloid matter—periosteum easily detached—and, at the point of fracture, where the spongy extremity narrowed into the shaft, the fragility was extreme.

The practical suggestions which occur to me from this case, are: First.—When there may be any election between resection and amputation, it would be advisable to make a more extensive convex external flap than usual; and, after articulation and eversion of the head of the bone, if the more formidable operation be deemed expedient, it can be expeditiously and easily completed by an internal flap.

Second.—As the disengagement of the head of the bone is frequently found difficult, and the cause of delay in operating by the ordinary method, might not the operation be generally conducted by cutting up and reflecting a large convex external flap, and after everting the head of the femur and adducting the thigh sufficiently to make the articulation start from its continuity upon division of the ligaments, complete the operation by thrusting the catling through to meet the posterior edge of first flap, and obtain sufficient covering by an internal flap?

